# School Gardening and Health and Well-Being of School-Aged Children: A Realist Synthesis

**DOI:** 10.3390/nu15051190

**Published:** 2023-02-27

**Authors:** Timothy P. Holloway, Lisa Dalton, Roger Hughes, Sisitha Jayasinghe, Kira A. E. Patterson, Sandra Murray, Robert Soward, Nuala M. Byrne, Andrew P. Hills, Kiran D. K. Ahuja

**Affiliations:** 1 School of Health Sciences, College of Health and Medicine, University of Tasmania, Launceston, TAS 7250, Australia; 2 School of Health Sciences, Swinburne University of Technology, Melbourne, VIC 3122, Australia; 3 School of Education, College of Arts, Law and Education, University of Tasmania, Launceston, TAS 7250, Australia; 4 Nutrition Society of Australia, Crows Nest, NSW 1585, Australia

**Keywords:** community gardens, school gardens, childhood education, experiential learning, nutrition, food security, childhood obesity, realist evaluation

## Abstract

School environments can create healthy settings to foster children’s health and well-being. School gardening is gaining popularity as an intervention for healthier eating and increased physical activity. We used a systematic realist approach to investigate how school gardens improve health and well-being outcomes for school-aged children, why, and in what circumstances. The context and mechanisms of the specific school gardening interventions (*n* = 24) leading to positive health and well-being outcomes for school-aged children were assessed. The impetus of many interventions was to increase fruit and vegetable intake and address the prevention of childhood obesity. Most interventions were conducted at primary schools with participating children in Grades 2 through 6. Types of positive outcomes included increased fruit and vegetable consumption, dietary fiber and vitamins A and C, improved body mass index, and improved well-being of children. Key mechanisms included embedding nutrition-based and garden-based education in the curriculum; experiential learning opportunities; family engagement and participation; authority figure engagement; cultural context; use of multi-prong approaches; and reinforcement of activities during implementation. This review shows that a combination of mechanisms works mutually through school gardening programs leading to improved health and well-being outcomes for school-aged children.

## 1. Introduction

Access to and consumption of healthy, nutritious food plays a crucially important role in maintaining good health and well-being and is a fundamental human right [1,2]. For many populations worldwide, however, deep-rooted and complex underlying problems associated with food systems influence the availability and access to healthy diets and nutritious food [2]. Food security exists when all people, at all times, have physical, social, and economic access to sufficient, safe, and nutritious food that meets both their dietary needs and food preferences for an active and healthy life [3]. Unfortunately, these conditions remain elusive for many [4], and in some instances, this leads to food insecurity. According to the Food and Agriculture Organization of the United Nations (FAO), the ability to be food secure largely depends on the uninterrupted supply and availability of different types of healthy food, food utilization, and the stability of each of these dimensions over time [3]. Additionally, a range of social determinants underpins the inequities in healthy eating [5]. For example, ‘urban poverty’, resulting from lower income availability, may lead to inadequate resources for people affected by such circumstances in accessing healthy diets, including fresh fruit and vegetables, and instead tend to consume higher quantities of sugars, fats, highly processed, and/or energy dense, ultra-processed foods [6].

Global urbanization and accompanying detachment from traditional agricultural practices have accentuated the decline in access to healthy food, including fruit and vegetables, and by extension, the associated nutritional benefits [7,8]. These dynamics are further complicated by the speed of transition to urban living and a simultaneous decline for some population groups in understanding healthy food production and consumption [7,8]. As a result, a plethora of public health interventions are geared towards increasing access to healthy, nutritious food. Community gardens, a space managed collectively by community members for growing food and non-edible plants [7,8,9], is a good example.

Community gardens are used in many settings, including residential neighborhoods, prisons, and schools [9]. Several scoping, narrative, systematic, and meta-analysis reviews suggest that school-based gardens are particularly useful in improving children’s nutritional outcomes [10,11,12,13,14,15]. For example, studies report that children’s fruit and vegetable consumption increased [13], and they were more willing to taste unfamiliar foods such as fruits and vegetables, cooking and food preparation skills improved, and nutritional knowledge increased [14]. Further, recent evidence also suggests health outcome improvements that transcend nutritional or food-related benefits, such as enhanced academic learning, social development, and improvements in general health and well-being [10,16]. As childhood obesity rates have increased dramatically over recent decades, school gardens have specifically been identified as settings to engage children in healthier eating and physical activity, with the objective of obesity prevention [15,17].

School gardening is widely reported to improve health and well-being outcomes [10,13,14,15,17,18]. However, systematic reviews report that quantitative evidence for changes in fruit and vegetable intake is limited and largely based on self-report [10] or limited through non-randomized study designs [13]. Although qualitative evidence reports a range of health and well-being benefits for school-aged children, these are rarely substantiated by quantitative evidence [10]. While more robust study designs would contribute to building the evidence base, using theory-led methods adds value by examining causal explanations of how and why school gardening interventions work [10]. This is the basis that we sought to address in this realist review.

The aim of the study was to assess the mechanisms which lead to positive health and well-being outcomes for school-aged children and answer the research question, “How do school gardens improve health and well-being outcomes for school-aged children?”

A systematic realist approach was selected for its value in moving beyond an investigation of “what works?” to focus on “how or why an intervention works, for whom, and in what circumstances?” [19]. Program theory guides the conduct of such systematic reviews, wherein reviewers seek to understand complex interventions [20,21,22].

## 2. Materials and Methods

### 2.1. Overview

Using a three-staged approach, the realist synthesis was used as the guiding methodology to analyze articles reporting school gardening interventions with positive outcomes.

The stages were to (1) identify relevant systematic, and meta-analysis review articles, (2) screen the Stage 1 reviews to extract primary source articles reporting positive health and well-being outcomes, and (3) use the primary source articles (from Stage 2) to identify specific school gardening interventions that robustly evidence health and well-being outcomes.

### 2.2. Searching the Literature and Defining Eligibility Criteria

Three databases (Scopus, Web of Science, PubMed) were systematically searched using the term, “school garden*”, which ensured broad coverage of the review articles (Stage 1). Inclusion criteria comprised peer-reviewed review articles only, published between 2012–2021 inclusive, and in English only. Exclusion criteria were applied to articles, book chapters, conference papers, proceeding papers, meeting abstracts, books and documents, clinical trials, and randomized controlled trials. Only systematic and meta-analysis reviews were included, and their search strategies had to clearly specify and adhere to The Preferred Reporting Items for Systematic reviews and Meta-Analyses (PRISMA) guidelines [23]. These review articles allowed for quick and efficient identification of primary sources/articles reporting on school gardening interventions.

### 2.3. Selection of School Gardening Reviews, Primary Articles, and Interventions

Identified review articles (Stage 1) were exported to EndNote reference management software (EndNote™ 20, Clarivate Analytics, Chandler, AZ, USA). Duplicate records were removed. Titles and abstracts were manually screened for terms related to “school garden/s” or “school gardening”, and articles were assessed for eligibility and inclusion.

Stage 2 included screening the full text of each eligible article to identify primary articles reporting positive health and well-being outcomes. Positive health and well-being outcomes were defined broadly as having improved change, either determined quantitatively (e.g., increased fruit and vegetable intake) or improved benefit determined qualitatively (e.g., improved behaviors towards fruit and vegetables). Positive health and wellbeing outcomes were identified from either text, tabulated data, or figure data. All study designs were identified, comprising quantitative, qualitative, and mixed-methods studies.

During Stage 3, the full text of each primary article was reviewed to identify specific school gardening interventions.

### 2.4. Data Extraction, Appraisal, Synthesis, Analysis, and Evaluation

Publication details, including authors, year of publication, location, objectives, study design, duration, participants, sample size, outcomes investigated, method of measuring outcomes, and details of positive health and well-being outcomes, were extracted from all included articles. To help improve the completeness in the reporting of the various interventions, the Template for Intervention Description and Replication (TIDieR) checklist and guidelines were used [24]. Data extraction was supplemented with key components: rationale, materials, procedures (activities), providers, delivery, timing, tailoring, modifications, and planning.

Data analysis drew on the principles of a realist synthesis for each school gardening intervention. This consisted of identifying the underlying causal or potential mechanism/s acting toward positive health and well-being outcomes by producing a Context–Mechanism–Outcome configuration for each of the school gardening interventions. If a number of primary articles were associated with a single intervention, then their data were combined during this Context–Mechanism–Outcome configuration process.

## 3. Results

### 3.1. Identification of School Gardening Interventions

**Figure 1:**
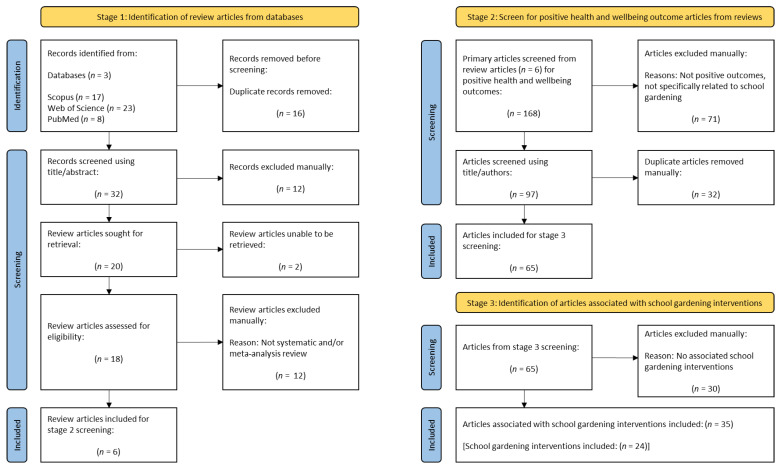
Three stages include identification of review articles, positive health and well-being articles, and articles associated with school gardening interventions.

Stage 1 screening identified 6 reviews for inclusion [10,13,14,15,17,18] (Figure 1; Supplementary Table S1); Stage 2 screening identified 65 primary articles with positive health and well-being outcomes; and Stage 3 screening identified 35 articles associated with 24 school gardening interventions [25,26,27,28,29,30,31,32,33,34,35,36,37,38,39,40,41,42,43,44,45,46,47,48,49,50,51,52,53,54,55,56,57,58,59].

### 3.2. Context–Mechanism–Outcome Configuration

For each intervention identified, a Context–Mechanism–Outcome configuration was developed, using the extracted data together with supplementary information from the TIDier process (Table 1).

#### 3.2.1. Context of School Gardening Interventions with Positive Health and Well-Being Outcomes

##### Location, Garden Spaces, and Facilitation

Identified school gardening interventions were conducted across a wide range of geographical locations, including Australia [25,26,27,28,29,30,31,32], the United Kingdom [33,34,35,57], the United States [36,37,38,39,40,41,42,43,44,45,46,47,48,49,50,51,52,53,54,55,56], India [57], Kenya [57], Bhutan [58], and Nepal [59] (Supplementary Table S2). Interventions mostly utilized gardens at school or child care premises, with the exception being community gardens or a summer camp garden [36,40,44]. Children and families participated in the design of gardens in interventions [27,29,30,57]. Initiatives were primarily facilitated by kindergarten, elementary, primary, and/or secondary school, and childcare center staff [25,26,27,28,29,30,31,32,33,34,35,37,38,39,40,41,42,43,44,45,46,47,48,49,50,51,52,53,54,55,57,58,59], with research teams [25,26,28,42,44,45,46,47,48,56], University departments [39,40], and external partners and/or specialists contributing in some contexts [25,26,29,30,31,32,33,34,35,36,37,38,39,40,41,42,43,44,45,46,47,48,49,53,54,55,56,58,59].

##### Rationale

School gardening interventions were predominantly used to influence school-aged children’s knowledge, attitudes, and/or behaviors toward diet and nutrition, particularly in connection to increasing fruit and/or vegetable consumption [25,26,29,34,35,36,37,38,39,42,43,44,45,46,47,48,50,51,52,53,54,55,56]. In many instances, this was associated with the impetus of addressing the prevalence and prevention of obesity [38,39,40,41,44,45,46,47,48,50,51,52,53,54,55], particularly as low-income minority groups may be disproportionately affected by lower fruit and/or vegetable intake and experience higher rates of childhood obesity [44,45,46,47,48,55]. Additionally, the ability of school gardens to influence physical activity and active living formed part of the reasoning for some interventions [38,42,55].

##### Participants and Activities

Most of the interventions were conducted at primary schools, with participating children in Grades 2 through 6. In multiple instances, nutrition and gardening education was integrated into the curriculum itself and delivered through school garden and kitchen activities [27,28,29,30,31,32,33,37,42,43,50,51,54,55,56,58,59]. Specifically, children were provided with opportunities to participate in growing, harvesting, and consuming garden produce (usually fruit and vegetables), with some enabling the sharing of meals together in a ‘family style’ environment [29,30,40,44,46]. Parental and family engagement were also encouraged through newsletters [25,26,36,40,52], take-home activities [36,37,55], and opportunities for volunteering [29,30,31,32]. Teacher training was also an important component in several interventions, particularly with nutritional and gardening activities [34,35,39,41,58,59]. Several interventions facilitated cultural awareness, including opportunities for cultural exchange or appreciation for culturally tailoring interventions in accordance with demographic profiles as focal points [27,40,44,45,46,47,48,55,57,58,59].

Some interventions were adapted from existing curricula, activity guides, peer-reviewed resources, or garnered from previous pilot initiatives. For example, several interventions were based on the curriculum of Junior Master Gardener^®^ (College Station, TX, USA) and Health & Nutrition from the Garden programs [37,43,54], and several utilized the activity guide developed by Lineberger and Zajicek (1998) [25,26,50,51]. Further, a few interventions were based on the model of Montessori (1964) and grounded in school gardening research and garden-based learning [49,57].

##### Duration, Frequency, and Type

Typically, the duration of interventions ranged from 6 weeks to 3 years [25,26,27,28,29,30,31,32,33,34,35,36,37,38,39,40,41,42,43,44,45,46,47,48,49,50,51,52,53,54,55,56,57,58,59]. “Frequency” and “type” of intervention also varied considerably and included a mix of weekly lessons (teaching nutrition, cooking, and/or gardening) [25,26,28,29,30,36,37,40,43,44,45,46,47,48,53,58,59], occasional expert/specialist visits [30,34,53,56], field trips [46,53], take-home activities [52,55], nutrition and cooking demonstrations and/or workshops [40,52], parental lessons [44,47,54], and teacher training sessions [34,35].

#### 3.2.2. Mechanisms Leading to Positive Health and Well-Being Outcomes

The combined action of nutrition-based and garden-based education, often integrated into the curriculum, was a common mechanism that contributed towards positive outcomes, particularly in connection to fruit and/or vegetables [25,26,33,44,45,46,47,48,50,51,58,59].

Experiential or “hands-on” learning experiences for students were also a common strategy amongst multiple interventions, with children involved in growing, nurturing, harvesting, preparing, and consuming produce from school gardens [25,26,27,29,30,31,32,36,44,45,46,47,48,49,50,51,55,57]. Reports also emphasized the effectiveness of experiential experiences as a pedagogical learning tool for students, with newly learned knowledge influencing attitudes, behavioral change, and building self-efficacy towards healthier eating [27,42].

The engagement and participation of families provided opportunities for intergenerational learning, informing behaviors and self-efficacy of children, and parents/guardians volunteering at school [27,29,30,31,32,36,37,49]. School teachers, principals, and other “authority figures” were important for behavioral modeling, leadership, and expertise as nutrition or gardening specialists [28,33,34,35,49,53,54]. Some interventions were tailored for minority groups, providing experiential learning opportunities in the context of cultural backgrounds and opportunities for intercultural learning [27,40,44,45,46,47,48,57,58,59].

A distinguishing feature was the use of multi-pronged approaches. For example, this included the adoption of multi-level and multi-sectoral methodologies, with involvement from individuals, community, and governmental agencies. In addition, programs implemented multi-component approaches including, for example, a combination of nutrition-based education, family involvement, development of community partnerships, support from the agricultural sector, and school wellness committees [33,39,40,41,44,45,46,47,48,52,53,58,59].

The reinforcement of activities leading to sustainability was also seen as a key mechanism, such as repeated and/or increased exposure to fruit and vegetables during the intervention duration. The notion of ensuring the impacts of school gardening activities was sustained was also accomplished by consistent and coordinated messaging through multiple intervention components [36,37,39,40,43,52].

#### 3.2.3. Positive Health and Well-Being Outcomes

Positive health and well-being outcomes were primarily related to fruit and vegetables (e.g., increased knowledge, awareness, preferences, behaviors, intake, and variety) [25,26,34,35,36,37,38,39,40,43,44,45,46,47,48,50,51,52,53,54,55,56,57,58,59]; dietary fiber, and vitamins A and C (e.g., increased intake) [44,45,46,47,48,50,51]; anthropometric measures (e.g., improved BMI percentile, BMI z-score, and waist-to-height ratio) [40,41,44,45,46,47,48,52,55]; children’s well-being (e.g., increased social skills and confidence, improved social connections, and a greater sense of belonging) [27,28,29,30,31,32]; and parent’s/family’s health and well-being (e.g., improved healthy eating, greater family interaction, and greater connection to school) [27,29,30,31,32,49].

## 4. Discussion

Through this realist synthesis, we investigated how school gardening improves health and well-being for school-aged children, finding that a combination of mechanisms operates in tandem under different contexts for the success of the school gardening interventions to yield positive outcomes. The impetus of many interventions was to increase fruit and vegetable intake and address the prevention of childhood obesity. Most were conducted at primary schools with participating children in Grades 2 through 6 and were located in high-income countries, including the United States and Australia. The mechanisms ranged from embedding nutrition and garden education in the curriculum to experiential learning, engagement and involvement of family and “authority figures”, and the relevance of cultural context. Types of positive outcomes included increased fruit and vegetable consumption, dietary fiber and vitamins A and C, improved BMI, and improved well-being of children.

The review results in evidence that the benefits of combining nutrition-based and garden-based education are important in improving outcomes, particularly with attitudes and behaviors toward fruit and vegetable consumption. This suggests that classroom-based lessons may be enhanced through practical and garden-based lessons. For example, in the How do you grow? How does your garden grow? intervention, the curriculum encompassed a variety of topics in relation to health and well-being, reinforced through ‘hands-on’ exposure to gardening activities [25,26]. Similarly, the Nutrition in the Garden program integrated nutrition education into the curriculum, with particular emphasis on a practical application involving comprehensive gardening and cooking activities [50,51]. In addition, findings from Berezowitz et al. (2015), through a review of school garden studies, conclude that garden-based learning may favorably affect fruit and vegetable consumption but also positively impacts academic performance [11]. Similarly, experiential learning strategies have proved useful in improving children’s knowledge, attitudes, and behaviors toward eating more healthily, including those in school garden settings [18]. Schools, therefore, have significant potential to create garden spaces for enabling experiential experiences linked to the curriculum, leading to enhanced learning and improved health and well-being outcomes.

Family involvement in school gardening initiatives was at the center of impacting positive health and well-being outcomes, demonstrated across several interventions, with mechanisms working at multiple levels. Previous research reports that family involvement helps change eating behaviors in school-aged children [14]. Consistent with the “bioecological theory” and “primary socialization theory”, a child’s development is collectively impacted by numerous proximal (e.g., parents, peers, community) and distal (e.g., cultural norms, laws, customs) influences and their complex interdependencies [60]. Accordingly, the importance of parents in promulgating healthy nutrition behaviors in children cannot be underestimated. Garnering the cooperation/participation of as many parents as possible in school-based gardening can be strengthened using volunteering programs and take-home activities, including produce and recipes. These strategies have proven to be effective at meaningfully engaging parents with school-garden-related activities [14].

Visionary leadership and inspirational role models are integral to school-based gardening interventions leading to health and well-being outcomes. Strong engagement between students and “authority figures”, including school teachers, school principals, and external experts, has consistently been shown to be associated with positive health and well-being outcomes. For example, Growing Schools and The Gloucestershire Food Strategy identified clear leadership and vision from the head teacher as critical for initiating change [33]. Findings from the Royal Horticultural Society Campaign for School Gardening indicate how the willingness of teachers to engage with the intervention may be important towards a greater intake of fruit and vegetables [35]. In addition, Viola (2006) identified how support from the school principal is key in the Outreach School Garden Project, leading to improved nutrition knowledge and skills [28]. More recently, Mann et al. (2022) synthesized evidence of nature-specific outdoor learning outside of the classroom on school children’s learning and development and suggested that all teacher training efforts should include skill development activities pertaining to this type of pedagogical approach [61]. Integration of ideas such as these is important as teachers are often highly influential during childhood education and development, as indicated above.

Considering the increasingly diverse societies we dwell in, it is no surprise that many made a conscious effort to accommodate the varying cultural needs in their interventions. For instance, culturally-tailored components, together with experiential learning, were central to the LA Sprouts program, leading to many potentially beneficial outcomes, including changed behaviors and preferences towards dietary fiber, fruit, and vegetables for children of Hispanic/Latino heritage [44,45,46,47,48]. Similarly, Ornelas and colleagues (2021) reported the importance of drawing on cultural strengths and traditional practices in addressing childhood obesity through school gardening, specifically for American Indian communities [62]. Therefore, cultural aspects and/or ethnic diversity would be an important consideration in the design of school gardening programs to ensure potential health and well-being outcomes are culturally sensitive and sustainable.

**Figure 2:**
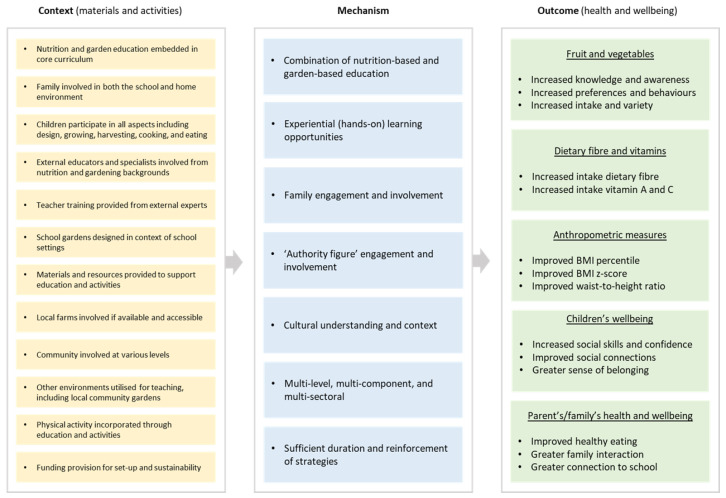
Context, mechanism, and outcome synthesis of school gardening interventions.

This realist review highlights that several key elements and numerous permutations of context and mechanisms work mutually, leading to positive health and well-being outcomes in school-aged children that may be observed collectively (Figure 2; Table 1). The synthesis demonstrates the potential for change when important contextual and mechanistic elements are drawn from a range of successful interventions that may be incorporated into current or proposed school gardening programs. This provides guidance in conjunction with published systematic and meta-analysis reporting on school gardening interventions. This also provides a template for consideration in designing new school gardening interventions or enabling adjustment and inclusion of additional elements to current interventions.

To the best of our knowledge, this is the first time a systematic realist synthesis with the accompanying use of program theory has been applied to school gardening interventions. The strength of this approach lies in using high-level research-based evidence through the identification of systematic and meta-analysis reviews. This informed identification of pertinent peer-reviewed primary articles with positive health and well-being outcomes and subsequent identification of school gardening interventions. This approach enabled the identification of evidence associated with school-based gardening interventions as previously identified and reviewed, allowing a comparison of our findings with the existing literature. Data extraction and TIDier checklist methodologies enabled holistic assessment of individual school gardening interventions, supporting robust configuration of context, mechanism, and outcomes and subsequent realist synthesis.

Notwithstanding the potential for positive outcomes that result from school gardens, it is important to note that the generalizability of the results from these interventions may be limited to high-income countries as most of the programs were based in Australia, the United Kingdom, and America. In addition, while a number of programs were based in areas of socio-economic disadvantage, addressing particular health inequities affecting low-income, under-resourced, and/or specific ethnic groups, including a focus on childhood obesity prevention, the results may not be entirely generalizable and transferable to other settings, either in other high-income countries or low-income countries.

## 5. Conclusions

Through this realist synthesis of identified school gardening interventions, we have shown how various mechanism work mutually to support positive health and well-being outcomes of school-aged children in particular contexts, which may assist with future endeavors. School gardening interventions potentially hold strong promise in supporting action toward the prevention of modern public health problems, including food insecurity and childhood obesity, both requiring urgent global attention.

## Figures and Tables

**Table 1:** Context–Mechanism–Outcome configuration of individual school gardening interventions.

Context (Materials/Activities)	Mechanism	Outcomes (Health/Well-Being)
How do you grow? How does your garden grow? [25,26] 10-week program with Grade 5–6 students		
“How do you grow?” nutrition education curriculum with topics on body, plants, nutrition, health, physical activity, and goal setting “How does your garden grow?” school garden component included the use of a garden and the production of a classroom cookbook Newsletters to encourage fruit and vegetable intake by families	Hands-on learning experience with garden-enhanced nutritional education with increased exposure to vegetables Some gender-specific factors. e.g., female teachers and female students performed better together, and girls socialized more in cooking and gardening	Higher willingness to taste vegetables and higher taste ratings of vegetables, especially peas, broccoli, tomato, and lettuce, in the intervention group
Multicultural School Gardens [27] 2-year program with 6–12-year-old children		
Integration of the program into the school curriculum Children and families (through the gardening buddies’ system) designed the garden, exchanged cultural activities, and learned English	Experiential learning through a “slow” pedagogical approach that provided intercultural and environmental learning opportunities, together with intergenerational experiences	Program enabled increased cultural awareness and sensitivity, increased sense of belonging and social connections, and fostered healthy eating habits
Outreach School Garden Project (OSGP) [28] 6-month project with Grades 5–6 and 7–9 students		
Nutrition extensively integrated into the school curriculum Teaching staff required no specific nutrition knowledge or gardening skills prior to the project Garden used to assist students with language, mathematics, measuring, problem-solving, writing skills, health and physical education, science and technology, and art and design	School principal key to supporting staff, students, and community Many core lessons able to be incorporated into the theme of garden and nutrition, thereby facilitating participation Garden acts as a catalyst for environmental action and change beyond the school	Positive improvements in student’s knowledge and skills in nutrition, gardening, and physical and social environment at school over a six-month period
Stephanie Alexander Kitchen Garden Program (SAKGP) [29,30,31,32] 2.5–3-year programs with Grade 3–6 students		
Children involved in all aspects, including garden design, planting, nurturing, harvesting, cooking, and sharing multi-course meals with specialist staff, teachers, and adult volunteers (often parents) Program provides professional development to educators, educational materials, and support Classes include a weekly 45 min garden class and 1.5 h kitchen class	Kitchen and garden experiences are enjoyable for the children Hands-on experiential and social learning with involvement in all aspects of garden design, planting, harvesting, and cooking Children exposed to a wide diversity of foods Motivations for volunteering, including belief in the program and desire to support school	Increased student engagement, social skills, and confidence Increase in children’s willingness to try new foods influencing healthy eating Volunteering by parents led to enhanced engagement between schools and the community, forming new friendships and relationships, leading to a sense of belonging and self-worth, and pride and pleasure in the community
Growing Schools and The Gloucestershire Food Strategy [33] 3-year programs with Grade 3 and Grade 6 students		
School gardening in a semi-rural primary school with emphasis on food and health in the curriculum Children participated in growing, harvesting, and eating vegetables from planters School used healthy caterers for the school menu	Leadership and vision (specifically, the head teacher) combined with community involvement (specifically, children, teachers, parents, and school governors) Accelerated and effective learning through critical thinking, practical hands-on approach, and decision-making, which helped students connect ideas to practice and provided motivation and a sense of ownership	Improvement in attitudes, awareness of health, and food Improvement in children’s eating habits
Royal Horticultural Society (RHS) Campaign for School Gardening [34,35] 1-year programs with Grade 3–4 students		
RHS-led intervention included visits by advisors to work with teachers and children, teacher training, and provision of free teacher resources versus a teacher-led intervention, with standard advice given by an RHS specialist for support in developing a school garden	Knowledge and attitudes mediate behavioral change towards fruits and vegetables Modeling and activity behavior of teacher toward fruit Teachers’ willingness to engage and their own gardening beliefs Teachers have daily contact with children	RHS-led intervention associated with a greater increase in total vegetables recognized Teacher-led group associated with higher intake of fruit and vegetables and willingness to taste new fruits
Delicious and Nutritious Garden [36] 12-week intervention with Grade 4–6 students attending a summer camp		
Garden plot designed and prepared Learning about plants and nutrition Growing, harvesting, and tasting fruit and vegetables Preparing healthful snacks Sharing experiences with family through newsletters and home-based activities	High value placed on the “seed to table approach” with hands-on activities, including planting, maintaining, harvesting, and preparing foods Repeated exposure to fruit and vegetables through taste tests, garden work, and snacks Children were agents of change in families through family involvement in home-based activities	Change in behavior (asking for fruit and vegetables at home) and increased intake of fruit and vegetables Increase in vegetable preferences Sense of ownership and pride in the garden
Eat Your Way to Better Health (EYWTBH) [37] 6–10-week program with Grade 3 students		
Lessons paired with Junior Master Gardener: Health and Nutrition from the Garden curriculum adapted to suit the needs of the school and community that facilitated experiential learning at school and take-home activities to do through the involvement of parents/guardians	Parents/guardians are seen as important environmental factors informing behavior and self-efficacy Greater ongoing fruit and vegetable consumption in those with previous diverse fruit and vegetable consumption	Improved healthy food choice self-efficacy and higher diversity of fruit and vegetable consumption
Gardens Reaching Our World (GROW) [38] 4.5-week program with Kindergarten to Grade 5 students		
Microfarm used as a gardening intervention, with students involved in growing, harvesting, and sampling microgreens Salad bar incorporated into the school cafeteria and presented to students as part of the school lunch program	Gardening lessons and activities may have enabled a greater quantity of vegetables selected from the salad bar	Increased consumption of vegetables per day during the intervention period Continued, but to a lesser degree, increase in vegetable consumption post-intervention
Got Dirt? Garden Initiative [39] 4-month initiative with 7–13-year-old students		
Small assistance grants provided to set up school gardens School gardens either in-ground, in containers, microfarms, or cold frame Training provided for teachers and early childhood providers with school gardens Children participated in gardening activities	Gardens impact in a socio-ecological systems manner, including intrapersonal, interpersonal, organizational, community, and policy levels Multi-layered impacts lead to cumulative effects and sustained behavioral change	Increased consumption of fruit and vegetables with students trying/tasting new fruit and vegetables (especially those grown in their garden) Choosing fruit and vegetables instead of chips or candy
Growing Healthy Kids (GHK) [40] 1-year program with 2–15-year-old children in the community		
Community gardens located at elementary schools, community parks, and privately-owned land Materials and tools provided along with weekly sessions to learn and practice gardening skills Workshops provided information and resources for making healthy food choices (also offered in Spanish for Hispanic families) Social events enabled whole family inclusion with dinners, meetings, garden construction activities, and newsletter production Gradually, families assumed responsibility for running activities and events	Community gardens appeal to newly-arrived immigrants by maintaining cultural traditions Continued access to community gardens with technical support and resources Families engage with the provision of nutritional classes Project able to influence policy change, enabling longer-term sustainability	Increased availability and consumption of fruits and vegetables among children of participating families Improvement in health as measured through Body Mass Index (BMI)
Healthier Options for Public Schoolchildren (HOPS)/The OrganWise Guys (OWG) [41] 2-year program with 4–13-year-old children		
Curriculum component designed; education and instructional material provided to teach children, parents, teachers, and other school staff about gardening, good nutrition, and healthy lifestyles Modifications made by dietitians to school-provided breakfasts, lunches, and snacks Increased physical activity opportunities made available during school time	School-based, multi-level, multi-sector approach Factors acting in concord, including dietary changes, nutrition education, and physical activity components	Significant improvements in BMI and blood pressure among low-income Hispanic and White children in the intervention group
Healthy Gardens, Healthy Youth [42] 2-year program with Grade 4–5 students		
Curriculum toolkit used based on extant garden curricula, including nutrition, horticulture, and plant science Educators led garden activities, including planting, weeding, harvesting, food safety, garden maintenance, engaging volunteers, capacity building, and program sustainability	Gardening-based lessons may be an effective pedagogical tool, facilitating a reduction in sedentary behaviors through movements including standing, kneeling, squatting, etc.	Higher moderate and vigorous physical activity, especially during outdoor garden-based lessons than during classroom-based lessons in the intervention group
Junior Master Gardener “Health and Nutrition from the Garden” [43] Up to 12-week programs with Grade 2–5 students		
Nutrition curriculum with material including activity guide “Health and Nutrition from the Garden” Delivery of activities (e.g., dietary fiber, budgeting, gardening, plant needs, healthy food pyramid, label reading, and food storage methods) varied according to location	Greater understanding of what should be eaten and why it should be eaten Nutrition curriculum effective at all ages, including younger and older children Nutrition curriculum enables a better understanding of food groups Increased exposure to fruit and vegetables through gardening activities	Significant improvement in knowledge regarding the benefits of eating fruit and vegetables Improved eating habits by eating healthier snacks after the nutritional program
LA Sprouts [44,45,46,47,48] 12-week programs with Grade 3–5 students		
Adaptive curriculum with culturally relevant focus, taught by an educator with a nutrition or gardening background Interactive, hands-on gardening activities plus cooking and nutrition education Raised garden in the community garden and school setting to provide parallel classes for parents and children Teaching conducted after-school on campus Meals prepared in small teams prepared vegetable/fruit snacks and shared in a family-style manner Monthly visits to local farmers’ markets integrated into the program Students encouraged to replicate recipes and conversations at home Successes and challenges documented by educators Project managers observed educator’s teaching to ensure adherence to the curriculum	Combination of culturally-tailored components and hands-on activities for gardening, cooking, and nutrition education that influenced attitudes, preferences, and motivations leading to increased knowledge and behavioral change Experiential learning, beginning with easy recipes to more complex recipes Affordability of home-grown foods Efficacious approach used to teach students to grow, prepare, and eat fruit and vegetables	Increased gardening and cooking attitudes, self-efficacy, motivation, and behavior associated with increased dietary fiber and vegetable intake and gardening at home Increased preference for vegetables, increased preferences for three target fruits and vegetables, and improved perceptions that “vegetables from the garden taste better than vegetables from the store” Fewer LA Sprouts participants had metabolic syndrome after intervention than before, while metabolic syndrome increased in controls Decreased diastolic blood pressure in LA Sprouts participants compared with the control group For overweight sub-sample: significant increase in dietary fiber intake, reduction in BMI, waist circumference, and less weight gain, compared to those in the control group
Master Gardener Classroom Garden Project [49] Ongoing project with Grade 2–3 students		
Garden plots available in schools Classroom gardens made available for teaching and guidance Support provided by Master Gardeners	Gardens enabled learning valuable moral lessons about life Hands-on experiences facilitated academic learning School gardening leads to greater home and family gardening, in turn leading to more active school participation Rewarding interactions leads to pleasant experiences Master Gardener was integral to the project	Positive effects on school children included gaining pleasure from observing the flourishing of garden products Children experienced increased interactions with parents/adults Children experienced the learning of emotions associated with harming things of value
Nutrition in the Garden [50,51] 1-year program with Grade 3–5 students		
Integration of nutrition education into curricula Use of activity guide ‘Nutrition in the Garden’ specifically relating to fruit and vegetables Thirty-four activities divided into 10 units, combining horticulture and nutrition subjects requiring the use of a garden or indoor grow lab and involving garden maintenance, salsa making, cooking classes, planting, harvesting, and consuming garden produce	Experiential exposure to fruit and vegetables builds self-efficacy and increased knowledge and awareness of nutrition Students with a greater need for improvement are more impacted Younger students more open to new ideas and experiences Females are more receptive to health and nutrition education and concerned about physical appearance	Improved students’ preferences and attitudes toward fruit, vegetables, and vegetable snacks Participating adolescents in garden-based intervention increased servings of fruit and vegetables more than control schools Significant increases in vitamin A, vitamin C, and fiber intake in experimental schools
Shaping Healthy Choices Program (SHCP) [52] 1-year program with Grade 4–5 students		
Curriculum comprised of five components: nutrition education and promotion, family and community partnerships, supporting regional agriculture, school food availability, and school wellness Activities included nutrition education, cooking demonstrations, school gardens, family newsletters, health fairs, salad bar implementation, procurement of regional produce, and school wellness committees	Major focus on consistent message reinforcement through lunchroom connections, community connections, and delivery at multiple venues Messaging coordinated throughout all program components, including growing, harvesting, and cooking Hands-on gardening and cooking activities enhanced the delivery of the curriculum	Greater improvement in BMI percentile, BMI z-score, and waist-to-height ratio in the intervention compared with control schools Significant improvements in nutrition knowledge and total vegetable identification in intervention schools
Sprouting Healthy Kids (SHK) [53] 5-month program with Grade 6–7 students		
In-class lessons comprised topics on healthy food, food production, and food security Farm-to-school component enabled locally grown vegetables to be served in the cafeteria Taste-testing of vegetables coincided with farmers’ visits, with encouragement to try different vegetables After-school visits to enable students to prepare and cook garden produce Farm visits to enable knowledge demonstration and assistance with farm tasks	Exposure to one intervention component sufficient to change knowledge regarding fruit and vegetables Behavioral and psychological change towards fruit and vegetables may come through a combination of activities, including exposure to two or more intervention components. e.g., ○ Interactive presentations by experts or “authority figures” such as farmers ○ Exposure to a greater variety of fruit and vegetables through taste testing ○ Provision of locally grown produce	Compared with students exposed to less than two intervention components, students who were exposed to two or more components scored significantly higher on fruit and vegetable intake, self-efficacy, and knowledge and lower on preference for unhealthy foods Although not significant, farmer’s visits, taste testing, and cafeteria components had the largest effect sizes
Texas Sprouts [54] 9-month intervention with Grade 3–5 students		
Raised vegetable beds, native herb beds, and large sheds for tools and materials built on school premises Nutrition curricula delivered by trained and paid nutrition and gardening educators Included preparation/cooking of fruit and vegetables, nutritious food choices, eating locally produced food, low-sugar beverages, health benefits of fruit and vegetables, eating healthfully in food desert neighborhoods, and food equity and community service	Lessons taught by well-trained and paid nutrition and gardening educators may be important (although not sustainable)	Increased vegetable intake in the intervention group
Texas!Grow!Eat!Go! (TGEG) [55] 4–6-month intervention with Grade 3 students		
Garden component included curricula centered around vegetables grown in the school garden Both garden and physical activity components included in the intervention School gardens constructed by AgriLife extension specialists, teachers, students, and parents Students grew vegetables, participated in both fresh vegetable sampling and recipe demonstrations, and take-home family activities	Experiential learning activities, including growing and harvesting vegetables, learning the benefits of eating vegetables, preparing simple vegetable recipes, and consuming food from recipes made at school	Improved nutrition knowledge, with an increase in vegetable preferences and vegetables tasted Decreased BMI percentile relative to children in comparison schools
Watch Me Grow [56] 4-month program with 3–5-year-old children at child care centers		
Raised beds installed at intervention childcare sites, with various fruit and vegetable crops grown and produce integrated with the center’s menu External health and gardening expertise provided Curricula modules and activities centered and delivered around each crop Published children’s books used to encourage connection to each crop	When vegetables are placed on plates for children to consume, this may lead to greater acceptance of vegetables	More vegetables served to; and more vegetables consumed by children in the intervention group
Gardens for Life (GfL) [57] 3-year project with 7–14-year-old students in different countries		
School gardens developed, tools supplied, with the growing of fruit and vegetables Children involved in the garden set-up Curriculum activities provided to improve understanding of fruit and vegetables, garden features and design, gardening activity and knowledge, and community and curriculum links	Experiential learning positively impacted curriculum learning in all settings, especially through improved self-esteem Mechanism may depend on culture and environment. e.g., English children viewed school gardens for pleasure, leisure, play, and enjoyment, where Indian and Kenyan children viewed school gardens for learning, community, security, and peace, while Indian children viewed school gardens in relation to conservation issues	Ten concepts developed to categorize outcomes, with generally highest scores recorded for knowledge on fruit and vegetables, gardening activity and knowledge, and curriculum and community links
Vegetables Go to School [58,59] 2-year program with Grades 6–7 students in different countries		
Curriculum used to teach students about gardening, nutrition, and WASH, with emphasis on “learning by doing” Project team taught teachers how to manage the school garden, with children cultivating nutrient-dense vegetables under the guidance of teachers, with parental support Promotional activities used to reinforce lessons and strengthen the impact	Linkage of school vegetable gardens to complementary lessons in agriculture, food and nutrition, and promotional activities Combination of gardening and education more effective than single components Collaboration and coordination among nutrition, health, and agricultural interventions	Significant increase in children’s awareness about fruit and vegetables, knowledge about sustainable agriculture, knowledge about food, nutrition, and health, and stated preferences for eating fruit and vegetables Increased probability that children included vegetables in their meals

Abbreviations: OSGP, Outreach School Garden Project; SAKGP, Stephanie Alexander Kitchen Garden Program; RHS, Royal Horticultural Society; EYWTBH, Eat Your Way to Better Health; GROW, Gardens Reaching Our World; GHK, Growing Healthy Kids; HOPS, Healthier Options for Public Schoolchildren; OWG, OrganWise Guys; SHCP, Shaping Healthy Choices Program; SHK, Sprouting Healthy Kids; TGEG, Texas!Grow!Eat!Go!; GfL, Gardens for Life.

## Data Availability

Not applicable.

## Supplementary Materials

The following supporting information can be downloaded at: https://www.mdpi.com/article/10.3390/nu15051190/s1, Table S1: Identification of school gardening articles with positive health and well-being outcomes; Table S2: Summary of school gardening interventions.
