# Supplementary material for: School Gardening and Health and Well-Being of School-Aged Children: A Realist Synthesis
Source: Nutrients. 2023 Feb 27;15(5):1190. doi: 10.3390/nu15051190 (PMC10005652; doi:10.3390/nu15051190)
Supplement: Supplementary file 1 [file nutrients-15-01190-s001.zip › Supplementary Table S1_TH.pdf]

nutrients

MDPI

**Supplementary Table S1.** Identification of school gardening articles with positive health and well-being outcomes.

| Review article | Aim | Identification of positive health and well-being outcome school gardening articles |
|---|---|---|
| Characteristics of successful primary school-based experiential nutrition programs: a systematic literature review [14]. | To identify key characteristics of successful experiential nutrition interventions aimed to change nutrition-related cognitive and behavioural outcomes in primary schoolchildren. | Number of articles reviewed: 42<br>• Articles included interventions involving school gardens, food provision, taste testing, cooking classes, and multicomponent programs.<br>Number of positive school gardening articles identified: 10<br>• 9 articles included school gardening,<br>• 1 articles included school gardening program as part of a multicomponent intervention, involving in-class lessons, after-school gardening, farm-to-school, farmer's visits, and farm field trips,<br>indicated positive health and well-being outcomes.<br>Reasons for exclusion of other studies:<br>• 32 studies excluded as articles not related to school gardening. |
| A systematic review of the health and well-being impacts of school gardening: synthesis of quantitative and qualitative evidence [10]. | To understand the health and well-being impacts of school gardens and the factors that help or hinder their success. | Number of articles reviewed: 40<br>• 21 quantitative studies,<br>• 16 qualitative studies, and<br>• 3 mixed-methods studies<br>Number of positive school gardening articles identified: 30<br>• 13 quantitative studies,<br>• 14 qualitative studies, and<br>• 3 mixed-methods studies,<br>indicated positive health and well-being outcomes.<br>Reasons for exclusion of other studies:<br>• 10 studies excluded as evidence indicated either neutral or no positive health and well-being outcomes. |

| | | |
|---|---|---|
| Is school gardening combined with physical activity intervention effective for improving childhood obesity? A systematic review and meta-analysis [17]. | To evaluate the effect of school gardening activities combined with physical activity on children's dietary intake and anthropometric outcomes. | Number of studies reviewed: 14<br>• 12 studies observed as being appropriate for meta-analysis.<br>• 2 studies included for review.<br>Number of positive school gardening studies: 13<br>• 13 studies indicated positive health and well-being outcomes.<br>Reasons for exclusion of other study:<br>• 1 study excluded as results for outcomes of interest were neutral. |
| School gardening activities aimed at obesity prevention improve body mass index and waist circumference parameters in school-aged children: a systematic review and meta-analysis [15]. | To analyze the main elements of school gardening with a specific meta-analysis about its impact on anthropometric parameters. | Number of studies reviewed: 33<br>• 33 studies included by authors as passing screening selection.<br>Number of positive school gardening studies: 28<br>• 28 studies indicated positive health and well-being outcomes.<br>Reasons for exclusion of other studies:<br>• 3 studies excluded as results were related to academic performance improvements only.<br>• 2 studies indicated no significant results in relation to health and well-being outcomes. |
| Increasing fruit and vegetable intake among children and youth through gardening-based interventions: a systematic review [13]. | To identify the effectiveness of gardening interventions that have been implemented to increase fruit and vegetable consumption among children. | Number of studies reviewed: 14<br>• 14 studies included with 10 studies demonstrating statistically significant increases in fruit and vegetable consumption among participants after implementation of a gardening intervention.<br>Number of positive school gardening studies: 12<br>• 12 studies included as they indicated positive outcomes whether or not they were statistically significant.<br>Reasons for exclusion of other studies:<br>• 2 studies excluded as evidence indicated either neutral or no positive health and well-being outcomes. |

| | | |
|---|---|---|
| Experiential learning interventions and healthy eating outcomes in children: a systematic literature review [18]. | To examine the effects of experiential learning activities on dietary outcomes (knowledge, attitudes, behaviors) in children. | Number of studies reviewed: 25<br>• 25 studies included by the authors with 9 types of experiential learning strategies used and observed as effective in improving nutrition.<br>Number of positive school gardening studies: 4<br>• 4 studies included as they involved school gardening as an experiential learning activity and indicated positive health and well-being outcomes.<br>Reasons for exclusion of other studies:<br>• 21 studies excluded as comprised experiential learning apart from school gardening, including taste-testing, games, creative/art studies, storybooks, shopping list development/food purchasing, food preparation/cooking, calculations/recording, and sensory evaluation. |
