# Supplementary material for: School Gardening and Health and Well-Being of School-Aged Children: A Realist Synthesis
Source: Nutrients. 2023 Feb 27;15(5):1190. doi: 10.3390/nu15051190 (PMC10005652; doi:10.3390/nu15051190)
Supplement: Supplementary file 1 [file nutrients-15-01190-s001.zip › Supplementary Table S2_TH.pdf]

nutrients MDPI

Supplementary Table S2. Summary of school gardening interventions.

Location	Description of intervention
Australia	**How do you grow? How does your garden grow?:** Nutrition education (How do you grow?) and gardening programme (How does your garden grow?) intervention, with previously used curricula and modified for the Australian context [25,26].
	**Multicultural School Gardens:** Program was instigated in disadvantaged and low-income schools and aimed to use food gardening for implementation of a culturally-focussed environmental education program [27].
	**Outreach School Garden Project (OSGP):** Project was conducted in Indigenous school communities and aimed to educate students on nutrition, founded on a simple and innovative approach with how the researcher learnt about nutrition as a child in the family garden and through home cooking [28].
	**Stephanie Alexander Kitchen Garden Program (SAKGP):** Program initiated in 2001 by Australian chef, restaurateur, and food writer Stephanie Alexander. Program provides a 'seed to table' experience, offering primary school aged children the opportunity to plant, nurture, harvest, prepare, and share fresh, nutritious, and seasonal food through gardening and kitchen based classes [29-32].
United Kingdom	**Growing Schools and The Gloucestershire Food Strategy:** Growing Schools is a government-funded programme with the aim to improve children's understanding of rural and urban environments, and their interdependence. The Gloucestershire Food Strategy is a programme implemented by the Gloucestershire Local Authority with the aim to promote patterns of healthy eating in schools [33].
	**Royal Horticultural Society Campaign for School Gardening:** Royal Horticultural Society (RHS) 'Campaign for School Gardening' enables work with schools to help children and young people experience gardening, through provision of resources, projects, and guidance [34,35].
United States	**Delicious and Nutritious Garden:** Pilot intervention designed to promote fruit and vegetable intake in children attending a YMCA summer camp, as informed by research-based evidence, and involving gardening based activities, including taste-testing of fruit and vegetables, healthy snack preparation, and family engagement [36].
	**Eat Your Way to Better Health (EYWTBH):** Program based on the Junior Master Gardener 'Health and Nutrition from the Garden' curriculum, including lessons on nutrition knowledge and application, experiential learning in the classroom garden, and engagement with parents/guardians [37].

	**Gardens Reaching Our World (GROW):** Program was a collaboration between Brown County University of Wisconsin-Extension's Community Garden Program and Green Bay Area Public Schools Food Service Department, using the school's salad bar in combination with introduction of a school gardening program [38].
	**Got Dirt? Garden Initiative:** A state-wide initiative led by the University of Wisconsin Extension in partnership with the Wisconsin Department of Health Services, aimed at improving children's health by increasing access to fruits and vegetables through youth gardening [39].
	**Growing Healthy Kids (GHK):** Program used community gardens as a means for providing low-income families with young children access to information regarding appropriate nutrition and healthy eating, including gardening sessions, cooking and nutrition workshops, and social events [40].
	**Healthier Options for Public Schoolchildren (HOPS)/The OrganWise Guys (OWG):** Program was a school-based obesity prevention intervention implemented in the elementary school setting, and included (1) modified dietary offerings, (2) nutrition and lifestyle educational curricula, (3) physical activity components, and (4) wellness projects [41].
	**Healthy Gardens, Healthy Youth:** Pilot program comprising four components: (1) raised garden bed for each class, (2) curriculum toolkit focused on nutrition, horticulture, and plant science, with accompanying gardening activities, (3) resources for the school, including information regarding food safety in the garden, and (4) garden implementation guide providing information regarding garden planning, planting and yearly maintenance; summer gardening; engaging volunteers; community capacity building; and sustaining the program [42].
	**Junior Master Gardener 'Health and Nutrition from the Garden':** Program aimed to teach children how to eat healthfully within limited budgets, consisting of six concepts, including (1) thrifty gardens, (2) basic gardening, (3) growing techniques, (4) ABCs of healthy eating, (5) healthy snacks, and (6) food safety, with each concept supported by six activities [43].
	**LA Sprouts:** A novel 12-week gardening, nutrition, and cooking program aimed at improving dietary intake and reducing obesity risk in Hispanic/Latino students, utilising school and community gardens [44-48].
	**Master Gardener Classroom Garden Project:** Project was established to provide inner-city children in the San Antonio Independent School District with an experiential way of learning about horticulture, gardening, themselves, and their relationships with fellow peers [49].
	**Nutrition in the Garden:** A garden activity guide developed to help teachers integrate nutrition education into their curricula. Activities in the guide are divided into 10 units including a total of 34 different activities, combining horticulture and nutrition subjects with detailed background information for teachers [50,51].
	**Shaping Healthy Choices Program (SHCP):** Program was a multi-component intervention designed for upper elementary school children with five overlapping components comprising: (1) nutrition education and promotion, (2) family and

	community partnerships, (3) supporting regional agriculture, (4) food availability on the school campus, and (5) school wellness committees and policies [52].
	**Sprouting Healthy Kids (SHK):** Intervention was garden-based and aimed to increase fruit and vegetable consumption, comprising six components: (1) in-class lessons, (2) after-school gardening program, (3) farm-to-school, (4) farmers' visits to schools, (5) taste testing, and (6) field trips to farms [53].
	**Texas Sprouts:** An intervention based on the pilot intervention LA Sprouts and Junior Master Gardener program developed by the Texas A&M AgriLife Extension Service, based on school gardening, nutrition, and cooking [54].
	**Texas!Grow! Eat!Go! (TGEG):** Intervention designed as a 2 x 2 factorial group randomized controlled trial in which 28 schools were randomly assigned to one of four conditions. The four conditions included (1) School Garden intervention (Learn!Grow!Eat!Go! [LGEG]), (2) Physical Activity (PA) intervention (Walk Across Texas program [WAT!]), (3) both Garden and PA interventions (Combined), and (4) neither a Garden nor PA intervention (Delayed Control) [55].
	**Watch Me Grow:** Program was garden-based and aimed to increase the number of vegetables and fruits provided for consumption by children in child care, through establishment of a fruit and vegetable garden, delivery of monthly curriculum, and provision of gardening support and technical assistance [56].
England, India and Kenya	**Gardens for Life (GfL):** An international project involving 67 schools in England, India and Kenya, focused on the growing of crops, and recognizing the importance of process and product of this activity in the included countries [57].
Bhutan and Nepal	**Vegetables Go To School:** Program was funded by the Swiss Agency for Development and Cooperation, with implementation conducted in collaboration with partners in Bhutan and Nepal [58,59].

Abbreviations: OSGP, Outreach School Garden Project; SAKGP, Stephanie Alexander Kitchen Garden Program; RHS, Royal Horticultural Society; EYWTBH, Eat Your Way to Better Health; GROW, Gardens Reaching Our World; GHK, Growing Healthy Kids; HOPS, Healthier Options for Public Schoolchildren; OWG, OrganWise Guys; SHCP, Shaping Healthy Choices Program; SHK, Sprouting Healthy Kids; TGEG, Texas!Grow!Eat!Go!; GfL, Gardens for Life.
